# T2 Magnetic Resonance Assay: Overview of Available Data and Clinical Implications

**DOI:** 10.3390/jof4020045

**Published:** 2018-04-04

**Authors:** Ioannis M. Zacharioudakis, Fainareti N. Zervou, Eleftherios Mylonakis

**Affiliations:** 1 Infectious Diseases Division, Warren Alpert Medical School of Brown University, Rhode Island Hospital 593 Eddy Street, Providence, RI 02903, USA; fainareti_zervou@brown.edu; 2 Department of Medicine, Warren Alpert Medical School of Brown University, Providence, RI 02912, USA

**Keywords:** candidemia, invasive candidiasis, invasive fungal infections, T2MR, PCR, molecular diagnostics

## Abstract

Invasive candidiasis is a common healthcare-associated infection with a high mortality rate that can exceed 60% in cases of septic shock. Blood culture performance is far from ideal, due to the long time to positivity and suppression by antifungal agents. The T2 Magnetic Resonance (T2MR) assay is an FDA-approved qualitative molecular diagnostic method that can detect and speciate the 5 most common *Candida* spp.; namely, *Candida albicans*, *Candida glabrata*, *Candida parapsilosis*, *Candida tropicalis*, and *Candida krusei*, in approximately 5 h. In a multicenter clinical trial that included both a prospective and a contrived arm to represent the full range of clinically relevant concentrations of *Candida* spp., T2MR demonstrated a sensitivity and specificity of 91.1% and 98.1%, respectively. The utility of T2MR in candidemia depends on the prevalence of disease in each clinical setting. In intensive care units and other high-prevalence settings, the incorporation of T2MR in diagnostic algorithms is very appealing. T2MR is expected to allow timely initiation of antifungal therapy and help with anti-fungal stewardship. In low-prevalence settings, the positive predictive value of T2MR might not be enough to justify initiation of antifungal treatment in itself. The performance of T2MR has not been studied in cases of deep-seated candidiasis. Despite some promising evidence in published clinical trials, further studies are needed to determine the performance of T2MR in invasive candidiasis without candidemia. Overall, experience with T2MR in everyday clinical practice is evolving but, in the right setting, this technology is expected to provide “actionable information” for the management of patients evaluated for candidemia.

## 1. Introduction

Invasive candidiasis is estimated to represent about 6% of all hospital-acquired infections, with approximately 46,000 cases diagnosed each year in the U.S. [1], and 250,000 cases worldwide, contributing to more than 50,000 deaths [2,3]. Invasive candidiasis encompasses a variety of clinical conditions that can be broadly divided into 3 categories: candidemia with no evidence of deep-seated candidiasis, candidemia with associated deep-seated candidiasis, and isolated deep-seated candidiasis without candidemia. Traditionally, candidemia, the most common form of invasive candidiasis, ranks as the third- or fourth-most common cause of health care-associated bloodstream infection, with a mean annual incidence of 13.3 per 100,000 population in Atlanta and 26.2 in Baltimore, as reported by the Centers for Disease Control and Prevention (CDC) in a U.S. population-based survey conducted in 2008–2011 [4].

Based on available evidence, primary candidemia can result in secondary deep-seated candidiasis in up to 50% of cases due to hematogenous seeding [5,6]. Additionally, direct seeding of tissues with *Candida* cells (e.g., in patients with recent abdominal surgery) may result in deep-seated candidiasis, which itself can result in secondary candidemia in approximately 20% of cases [7]. Use of broad-spectrum antibiotics, critical illness, presence of central venous catheters (CVCs), total parenteral nutrition (TPN), immunosuppression, mechanical ventilation, renal insufficiency, hematologic and solid organ malignancy or transplantation are the main risk factors for invasive candidiasis [2].

As invasive candidiasis includes a number of clinical conditions in a variety of hosts, the mortality associated with this infection varies significantly. In a meta-analysis of randomized clinical trials that included patients with all forms of invasive candidiasis, Andes et al. calculated that the 30-day mortality in invasive candidiasis to be 31.4%, with a range among individual studies of 20–40%. It should be noted that in this meta-analysis, more than 80% of the infections were bloodstream infections, and that the included randomized trials were designed for medication approval, so not all patients were treated with current first-line antifungal therapy [8]. In patients with septic shock secondary to candidemia, the mortality rate can exceed 60% [9]. Early initiation of appropriate antifungal therapy is a major determinant of prognosis, together with source control, particularly among patients with septic shock [9,10,11]. For example, in a study by Garey et al. the mortality rate was 14.1% when treatment was started at the day of blood culture draw, 23.5% when it was started 1–2 days after blood culture draw and 42.3% thereafter [10]. Based on the significance of timely initiation of antifungal therapy, the development of minimally invasive, highly sensitive and specific diagnostic tests with a quick turn-around time and reasonable cost have the potential to significantly improve outcomes.

## 2. Current Diagnostics and Limitations

Culture of blood and other tissue samples have historically been considered the gold standard for diagnosis of invasive candidiasis and remains the only method that allows for sensitivity testing of the isolated *Candida* spp., making culture an essential component of every diagnostic algorithm. Blood culture can detect candidemia at inocula as low as 1 colony-forming unit (CFU)/mL, which is lower than the concentration usually required for reliable detection of candidemia by polymerase chain reaction (PCR) assays [12]. However, the utility of blood cultures in the diagnosis of invasive candidiasis is far from excellent, with pre-mortem blood cultures being positive in 21 to 71% of patients with later autopsy-proven invasive candidiasis [5,13,14].

There are intrinsic characteristics of blood cultures that make them unattractive as the only test used for the diagnosis and guidance of treatment in invasive candidiasis. By definition, blood cultures are not useful in diagnosing isolated deep-seated candidiasis caused by direct inoculation of tissues with *Candida* cells without secondary candidemia. Additionally, the presence of antifungal agents suppresses the yield from blood cultures [15,16]. For example, in a study that examined the performance of two commonly used blood culture systems, the addition of therapeutic levels of antifungal agents in seeded blood culture bottles decreased the detection rate of *Candida* spp. from >95% to 50–83% [17]. This becomes particularly important when examining immunocompromised patients on prophylactic antifungal agents. For example, Kami et al. examined 720 cases with hematologic malignancy on fluconazole prophylaxis and found that only 20 among the 94 patients with autopsy-proven invasive candidiasis had a positive blood culture before death [14].

The slow turn-around time of blood cultures is another limiting factor in the diagnosis of invasive candidiasis and becomes of utmost importance in septic patients, where there is an increasing mortality rate with every hour delay in initiation of effective treatment. For example, Kollef et al. examined patients with septic shock associated with the isolation of *Candida* spp. in blood cultures and found that approximately 20% of patients died before blood culture turned positive, and before the administration of the first dose of antifungals [9]. Time to positivity differs among *Candida* spp., with *Candida glabrata*, for example, having a significantly longer incubation period compared to *Candida tropicalis* [18,19]. In a study comparing BACTEC Plus Aerobic/F blood cultures and T2 Magnetic Resonance (T2MR) assay, 0% (0/20) of the seeded blood culture bottles with *C. glabrata* was positive in 5 days with the BACTEC blood culture system, in comparison to 100% with the T2MR assay [20]. The above is particularly important in low-level breakthrough candidemia in hematologic patients that is often caused by *C. glabrata* [21]. Finally, the slow turn-around time of blood cultures makes them a suboptimal tool for evaluating the mycologic response to therapy [22].

In light of the significant morbidity and mortality associated with invasive candidiasis, and to overcome the shortcomings of blood cultures, continuous efforts are made to develop and evaluate molecular and other non-culture diagnostic tests as adjuncts to blood cultures for the diagnosis and treatment of invasive candidiasis. A diagnostic marker, the β-d-glucan assay (BDG) (Fungitell; Associates of Cape Cod, East Falmouth, MA, USA) has been approved by the U.S. Food and Drug Administration (FDA) as an aid for the diagnosis of invasive candidiasis and is currently implemented in daily clinical practice. Multiple studies have been performed to evaluate the performance of this test in guiding empiric antifungal administration, and its utility in monitoring response to treatment. In a bivariate meta-analysis of 8 studies, BDG was found to have a sensitivity of 77.9% for invasive candidiasis among different at-risk populations [23]. However, as the BDG is a major component of the cell wall of different fungi including *Candida* spp., *Aspergillus* spp., *Furarium* spp., as well as *Pneumocystis jiroveci*, it cannot distinguish between different fungal infections and does not provide *Candida* speciation. As such, in 2 additional meta-analyses, pooled sensitivity for the diagnosis of all invasive fungal infections was reported at 76.8% [24] and 80% [25], with a specificity of 85.3% and 82% respectively. However, a high heterogeneity has been observed regarding the performance of BDG in individual studies [23].

In a study among oncology and hematology patients, the sensitivity and specificity of BDG at the time of diagnosis of invasive candidiasis was 43.5% and 73% [26], while the corresponding numbers in the surgical intensive care unit were 87% and 73% [27]. Posteraro et al. compared the performance of BDG with that of *Candida* score and colonization index in septic patients with >5 days length of stay in an intensive care unit (ICU) [28]. The positive (PPV) and negative predictive values (NPV) for the BDG were higher than those of the *Candida* score and colonization index (PPV 72.2% vs. 57.1% and 27.3%; NPV 98.7% vs. 97.2% and 91.7%, respectively) [28]. Notably, BDG has demonstrated different sensitivities among *Candida* spp., with reported 72% sensitivity for *Candida albicans* and 41% for *Candida parapsilosis* [29,30]. The number of samplings and the cut-offs used also affect BDG performance [24,31]. Finally, multiple factors, as for example, the cellulose based dialysis filters have been associated with false positive results [32].

Despite the limitations of BDG as a diagnostic tool for invasive candidiasis, it has a unique characteristic when used for monitoring the response to therapy due to its quantitative profile [26]. A negative slope in BDG levels correlates with a successful treatment outcome with a PPV of 90%, while a positive slope correlates with treatment failure with a negative predictive value (NPV) of 90% [33,34]. BDG has been used in algorithms to guide early discontinuation of empiric antifungal agents. In a multicenter trial, patients who were considered high risk for invasive candidiasis based on clinical prediction rules had BDG and blood cultures drawn on days 1 and 3 while on empiric anidulafungin. Anidulafungin was discontinued in patients with negative results. By day 4, 25% of patients were off antifungals and none of them was diagnosed with candidemia by day 30 [35].

Apart from the BDG, other non-culture diagnostic methods exist for the diagnosis of invasive candidiasis [36], including the mannan antigen and the anti-mannan antibody [37], the *C. albicans* germ tube antibody (CAGTA) [38], and various molecular assays [39]. In a meta-analysis of 14 studies by Mikulska et al., which included hematologic and ICU patients, the sensitivity and specificity of a combined approach that used mannan and anti-mannan testing was 83% and 86%, respectively, with the best performance among patients with *C. albicans* infection [40]. CAGTA detects antibodies against the surfaces of *C. albicans* germ tubes with sensitivity and specificity ranging from 77–89% to 91–100% respectively [41], and has been shown to have a role in detecting deep-seated candidiasis in candidemic patients [42]. The rapid turn-around of PCR assays, and the identification of the microorganism at a species level make their use appealing for early diagnosis, guidance of antifungal therapy, and monitoring of mycologic response to therapy. However, the lack of validation and standardization has limited the implementation of PCR in daily clinical practice. The comparative sensitivity and specificity of different PCR assays was studied in a meta-analysis of 54 studies by Avni et al. [43]. Despite the promising reports, a big variation in PCR performance was noticed depending on the specimen type (serum, whole blood and plasma), the PCR gene target, and the number of PCR tests performed to define the presence or not of the infection. The authors of this meta-analysis concluded that whole blood PCR using pan-fungal primers might result in better diagnostic accuracy, but conflicting reports from individual studies have also been published [44]. Finally, the MALDI-TOF MS assay, using mass spectrometry to identify the protein fingerprints of different organisms, is currently approved by FDA for use in microbiology labs [45,46] and can decrease the time to species identification [47], but requires positive blood cultures [48].

## 3. T2MR Assay

In 2014, the FDA approved the T2MR assay, a fully automated qualitative assay run on the T2Dx instrument, for the diagnosis of candidemia. T2MR combines the nuclear magnetic resonance and PCR molecular assay to directly detect and define the species of *Candida* spp. from whole blood samples. Specifically, T2Dx lyses the *Candida* cells by mechanical bead beating and amplifies the *Candida* DNA using a thermostable polymerase (T2Biosystems, Inc., Wilmington, MA, USA) and pan-*Candida* primers for the intervening transcribed spacer 2 region within the *Candida* ribosomal DNA operon. T2MR can detect 5 *Candida* spp., namely *C. albicans*, *C. glabrata*, *C. parapsilosis*, *C. tropicalis*, and *Candida krusei*. T2MR cannot differentiate though the species of *C. parapsilosis* complex, namely *C. parapsilosis*, *C. orthopsisllosis* and *C. metapsillosis*. The speciation is achieved through hybridization of capture probes specific for the above species within the pan-*Candida* amplicons [49]. The amplified DNA is detected by hybridization to the superparamagnetic nanoparticles, which causes large changes in the sample’s T2MR signal. The T2MR assay reports the results as follows: *C. albicans*/*C. tropicalis*, *C. krusei*/*C. glabrata*, and *C. parapsilosis*. To monitor the integrity of the T2MR results, an internal control, which is a synthetic DNA target, is processed with each clinical specimen. If the internal control is invalid and there are no positive T2MR signals, an “invalid” result is displayed, and indicates that the specimen might contain inhibitors that would interfere with the detection of *Candida* spp. The materials needed and the methods used to diagnose candidemia using the T2MR assay are summarized in [50].

## 4. Performance of T2MR

**Figure 1:**
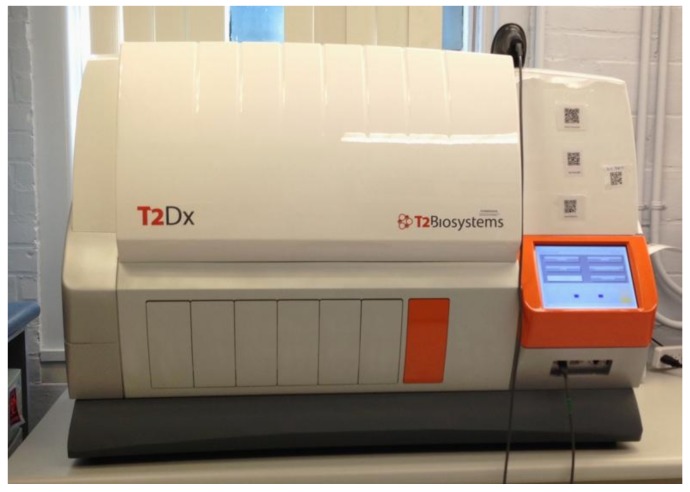
T2Dx Instrument.

The performance of the T2MR assay was first studied with a small portable T2MR detection device on spiked whole-blood samples by Neely et al. [49]. T2MR was found to have a limit of detection of 3 CFU/mL for *C. albicans* and *C. tropicalis*, 2 CFU/mL for *C. krusei* and *C. glabrata* and 1 CFU/mL for *C. parapsilosis* [49]. No impact on the detection, sensitivity, and reproducibility was observed when blood from patients with suspected sepsis was used. Additional interference studies for 25 endogenous and exogenous substances, including 11 antibacterial and antifungal agents, indicated no significant change in assay performance. In that study, a 98% positive agreement and 100% negative agreement were observed between the T2MR and blood cultures in in vitro-spiked healthy donor whole-blood specimens. The aforementioned promising results, and the time to result of ~2 h, led to the development of the fully automated T2Dx instrument, which completes all steps of the T2MR assay after specimen loading (Figure 1).

An extensive clinical trial was conducted by Mylonakis et al. to validate the sensitivity and specificity of the T2MR assay compared to blood cultures [51]. The study included both a prospective and a contrived arm in order to have representation of the full range of clinically relevant concentrations of *Candida* spp. For the prospective component of the trial, 1501 patients, who had a blood culture ordered per routine standard of care during their hospitalization, were enrolled. A total of 300 whole blood specimens were also used, 250 of which were manually supplemented with clinically relevant titers of the 5 *Candida* spp. targeted by the T2MR, and 50 were used as negative controls. The T2MR assay was found to have an overall sensitivity of 91.1%, with sensitivity of 92.3% for *C. albicans*/*C. tropicalis*, 94.2% for *C. parapsilosis*, and 88.1% for *C. krusei*/*C. glabrata*. The overall specificity of T2MR was found to be 98.1% with the mean time to result being approximately 5 h. The limitation that T2MR can detect only 5 *Candida* spp. was noted, especially given the current shift in the *Candida* spp. epidemiology. However, the 5 *Candida* spp. targeted by the T2MR still represent >50% of cases of candidemia.

Another recently published multi-center clinical trial by Clancy et al. came to validate the clinical sensitivity of T2MR in candidemic patients [52]. In this trial, 152 patients with candidemia, as detected by blood cultures, were enrolled and had follow-up T2MR samples and companion blood cultures collected at a median time of 55.5 h from the initial diagnostic blood cultures. By the time of follow-up testing, 74% of the enrolled patients had received at least 1 dose of antifungal therapy. Both T2MR and companion blood cultures were negative in 52% of cases. T2MR was found to have a sensitivity 88.9%, as positive results were obtained in 32/36 of patients with positive companion blood cultures. It is worth noting that T2MR positive results with negative companion blood culture were obtained in 24% of cases (37/152 patients). Prior antifungal treatment was associated with T2MR positive/culture negative results [52]. This was not surprising as it is known that T2MR, in contrast to blood cultures, can detect growth-inhibited *Candida* cells. Both studies had a significant number of invalid results: 7.6% [51] and 9.2% [52], respectively. While these may result from the presence of inhibitors, as described above, no risk factors have been identified so far.

Hamula et al. studied further the performance of T2MR in the pediatric population. A total of 15 children aged 5–12 years old with candidemia and 9 without were studied [53]. A new loading method with pipetting whole blood directly onto the T2Candida cartridge was developed to minimize the required amount of blood. T2MR identified correctly all candidemic and non-candidemic patients with appropriate *Candida* speciation, indicating that the T2MR performs equally well in low-volume pediatric blood specimens.

## 5. Utility of T2MR in Culture-Positive and Culture-Negative Invasive Candidiasis

The available evidence regarding the performance of the T2MR assay makes the incorporation of T2MR in the diagnostic algorithms of severe sepsis and septic shock in high prevalence settings very appealing. In an ICU setting with a prevalence of candidemia of 10%, T2MR is expected to have a positive and negative predictive value of 84.2% and 99%, respectively [51]. As this performance has been estimated in comparison to the current gold standard diagnostic method—the blood cultures—these results mean that in 84.2% of cases with a positive T2MR result, the accompanying blood culture will also grow *Candida* spp. On the other hand, in 99% of cases with a negative T2MR result, the blood culture will also be negative.

Other high-risk patient populations, such as hematologic patients, patients with febrile neutropenia, hematologic stem cell transplant recipients, solid organ transplant recipients with sepsis, end-stage renal disease patients on hemodialysis or patients on total parenteral nutrition, might also benefit from T2MR testing at the time of blood culture draw. For example, febrile neutropenia in patients with hematologic malignancies on chemotherapy or after bone marrow transplantation who are not on empiric antifungal agents has been shown to be secondary to *Candida* bloodstream infection in 1.5–3.2%, which would correlate with a T2MR PPV of 42.2–61.3% [54,55,56].

In contrast, in the general hospital setting, the prevalence of candidemia is significantly lower. In the multicenter trial noted above, in which T2MR was collected, along with blood cultures as per routine practice, *Candida* spp. was isolated in 6 out of 1501 patients for a prevalence of 0.4%. In such a low-prevalence setting, the positive predictive value of a positive T2MR is estimated to be 15%, and a positive result would be unlikely by itself to justify antifungal treatment in a patient without identifiable risk factors for candidemia. Therefore, in such low-prevalence settings, an overall risk stratification of patients is required, based on their risk factors [2].

Clinical outcomes from the incorporation of T2MR in algorithms of diagnosis and treatment of candidemia are limited. In a recent retrospective study, incorporation of T2MR in the diagnostic algorithm for candidemia, was able to reduce the mean time to appropriate antifungal therapy from 40 h prior to implementation to 27 h [57]. Additionally, the performance of T2MR assay as a monitoring tool of the mycologic response to antifungal therapy was recently evaluated in a multicenter prospective clinical trial, the STAMP trial [22]. The study included 31 patients who had been diagnosed with candidemia and had been started on antifungal therapy. Patients had serial blood cultures and T2MR assays drawn in pre-specified intervals to monitor candidemia clearance [58]. By the end of the first surveillance week, candidemia was still detected in 15.4% of patients by T2MR vs. 0% by the blood cultures with an overall statistically significant improvement of post-treatment surveillance with the T2MR assay. Possible explanations for the observed difference were thought to be the intermittent nature of candidemia in deep-seated infections, the inhibition of blood cultures by therapeutic levels of antifungal agents, and the low level of candidemia when disease arises from the gastrointestinal tract, especially with *C. glabrata*, which, as discussed above, may also need more than the traditional 5-day incubation period to grow. Based on this study [22], it is reasonable to assume that persistently positive T2MR results might provide actionable results in daily clinical practice to guide further diagnostic and therapeutic interventions, such as removal of CVCs in patients in whom salvage was initially attempted.

While candidemia is a clearly defined clinical entity, culture-negative invasive candidiasis encompasses a wide variety of clinical conditions with different risk factors, treatment strategies, and prognosis. Culture-negative invasive candidiasis includes deep-seated candidiasis that arises from direct inoculation of sterile tissues with *Candida* cells, or from prior hematogenous spread of *Candida* spp., whether this was captured by blood cultures or not. The role of T2MR in early diagnosis of the culture-negative invasive candidiasis infections is not clear given that there are no studies involving T2MR in the diagnosis of the above clinical entities. However, there are a few data in the DIRECT2 clinical trial and the STAMP trial that could support that T2MR can detect at least some cases of deep-seated candidiasis [22,51]. For example, in the DIRECT2 trial there was a case with positive T2MR and more than 12 negative blood cultures who was found to have intra-abdominal *C. albicans* infection, as was proven later with peri-operative tissue cultures [51]. Additionally, in the STAMP trial, a patient had a positive T2MR result 5 days after study enrollment, followed by 2 negative surveillance T2MR specimens, and persistently negative surveillance blood cultures that correlated with the diagnosis of *C. albicans* biloma [22]. However, these limited data are not enough to justify the utility of T2MR in deep-seated candidiasis and should be studied in future trials. Further, it is unclear if a positive T2MR result, discordant with the blood cultures, should prompt work-up to evaluate for deep-seated candidiasis without candidemia.

## 6. Conclusions

In conclusion, T2MR represents a highly promising molecular diagnostic method that allows the rapid, accurate, and species-specific diagnosis of candidemia. The positive T2MR results should be interpreted in the context of the expected prevalence of the disease in the specific clinical setting. We should note the paucity of data regarding the cases with culture negative invasive candidiasis and the evolving everyday clinical experience with this new technology. Future studies remain to determine the performance of T2MR in patients diagnosed with deep-seated infections by following those patients with T2MR, blood cultures and fungal markers. Studies should also examine the clinical outcomes and costs when T2MR is implemented to guide the initiation and/or the duration of anti-fungal therapy, especially in settings with expected high prevalence of the disease and high mortality.
